# Biofilm formation assessment in *Sinorhizobium meliloti* reveals interlinked control with surface motility

**DOI:** 10.1186/s12866-015-0390-z

**Published:** 2015-03-03

**Authors:** Carol V Amaya-Gómez, Ann M Hirsch, María J Soto

**Affiliations:** Departamento de Microbiología del Suelo y Sistemas Simbióticos, Estación Experimental del Zaidín, Consejo Superior de Investigaciones Científicas (CSIC), Profesor Albareda 1, 18008 Granada, Spain; Department of Molecular, Cell, and Developmental Biology and Molecular Biology Institute, University of California-Los Angeles, Los Angeles, CA 90095-1606 USA

**Keywords:** FadD, Iron, *Rhizobium*, RirA, Root colonization, Siderophore, Swarming

## Abstract

**Background:**

Swarming motility and biofilm formation are opposite, but related surface-associated behaviors that allow various pathogenic bacteria to colonize and invade their hosts. In *Sinorhizobium meliloti,* the alfalfa endosymbiont, these bacterial processes and their relevance for host plant colonization are largely unexplored. Our previous work demonstrated distinct swarming abilities in two *S. meliloti* strains (Rm1021 and GR4) and revealed that both environmental cues (iron concentration) and bacterial genes (*fadD*, *rhb*, *rirA*) play crucial roles in the control of surface motility in this rhizobial species. In the current study, we investigate whether these factors have an impact on the ability of *S. meliloti* to establish biofilms and to colonize host roots.

**Results:**

We found that strain GR4, which is less prone to translocate on solid surfaces than strain Rm1021, is more efficient in developing biofilms on glass and plant root surfaces. High iron conditions, known to prevent surface motility in a wild-type strain of *S. meliloti*, promote biofilm development in Rm1021 and GR4 strains by inducing the formation of more structured and thicker biofilms than those formed under low iron levels. Moreover, three different *S. meliloti* mutants (*fadD, rhb*, and *rirA*) that exhibit an altered surface translocation behavior compared with the wild-type strain, establish reduced biofilms on both glass and alfalfa root surfaces. Iron-rich conditions neither rescue the defect in biofilm formation shown by the *rhb* mutant, which is unable to produce the siderophore rhizobactin 1021 (Rhb1021), nor have any impact on biofilms formed by the iron-response regulator *rirA* mutant. On the other hand, *S. meliloti* FadD loss-of-function mutants do not establish normal biofilms irrespective of iron levels.

**Conclusions:**

Our studies show that siderophore Rhb1021 is not only required for surface translocation, but also for biofilm formation on glass and root surfaces by strain Rm1021. In addition, we present evidence for the existence of control mechanisms that inversely regulate swarming and biofilm formation in *S. meliloti*, and that contribute to efficient plant root colonization. One of these mechanisms involves iron levels and the iron global regulator RirA. The other mechanism involves the participation of the fatty acid metabolism-related enzyme FadD.

**Electronic supplementary material:**

The online version of this article (doi:10.1186/s12866-015-0390-z) contains supplementary material, which is available to authorized users.

## Background

Swarming motility and biofilms are two different and opposite behaviors displayed by bacteria living on surfaces. Biofilms are sessile assemblages of microorganisms embedded in a self-produced polymeric matrix that adhere to a surface or are associated with interfaces [1,2]. By contrast, swarming is a mode of surface translocation that depends on rotating flagella and is characterized by the rapid and coordinated movement of multicellular groups of bacteria [3]. Several studies have revealed the existence of a link between swarming and biofilm formation: i) both are surface-associated multicellular processes in which cell-cell communication and quorum sensing play important roles; ii) in both processes, the participation of the same cell surface-associated structures such as flagella, a polysaccharide matrix, and biosurfactants has been reported; and iii) swarming bacteria, similar to bacteria in biofilms, show increased resistance to several antimicrobial agents [3-8]. Studies performed in *Pseudomonas aeruginosa*, *Vibrio cholerae*, or *V. parahaemolyticus* show that the two lifestyles are inversely regulated by a common pathway, which is modulated by the intracellular second messenger cyclic di-GMP [9-14].

Swarming motility and biofilm formation have been studied almost exclusively in pathogenic bacteria. However, little is known about these multicellular surface-associated responses in rhizobia, soil-dwelling bacteria, which induce nitrogen-fixing nodules on the roots of legume plants following a complex and continuous molecular dialogue that co-ordinates bacterial infection with nodule organogenesis [15].

*Sinorhizobium meliloti*, the alfalfa symbiont, forms biofilms on both abiotic surfaces and roots [16]. On abiotic surfaces, the capability of *S. meliloti* to form biofilms is affected by environmental stresses and nutrient status [17]. As in many bacteria, rhizobial exopolysaccharides (EPS) and flagella are involved in biofilm formation and mutants defective in either of these two components exhibit a significant reduction in the ability to develop biofilms [16,18-20]. Remarkably, the production of a low-molecular-weight fraction of galactoglucan (EPS II), the production of which is dependent on a functional ExpR/Sin quorum sensing system, is crucial for biofilm formation and root colonization. EPS II-producing strains are able to develop highly structured biofilms under low-phosphate conditions, but not under high phosphate conditions where flat and unstructured biofilms are formed [18]. Besides EPS and flagella, core Nod Factor, an essential molecule for the nodulation process, has been shown to be critical for biofilm formation in *S. meliloti* [21]. In addition to the LuxR-type transcriptional regulator ExpR, different regulatory proteins that control several phenotypes including EPS production and motility have been involved in regulation of biofilm formation in *S. meliloti*. This is the case for the two-component system ExoS/ChvI and its periplasmic inhibitor ExoR [16,22]; or EmrR, a TetR-family transcriptional repressor that controls expression of *emrA* and *emrB*, which encode a putative MFS-type transporter [23].

Swarming motility has also been reported in *S. meliloti* [24-27], and was first described for a *fadD* mutant of the GR4 strain [24]. Wild-type GR4 cells normally do not translocate over semisolid surfaces, but inactivating the *fadD* gene, which codes for a long-chain fatty acyl-coenzyme A ligase, promotes swarming motility on semisolid minimal medium. This finding strongly suggests that FadD plays a role in the control of this multicellular surface-associated behavior. However, in contrast to GR4, the commonly used *S. meliloti* laboratory strain Rm1021 moves over semisolid surfaces using flagella-dependent and -independent mechanisms [25,26]. The fact that wild-type GR4 cells do not translocate in contrast to Rm1021 cells and that a mutation in the *fadD* gene promotes surface translocation for both strongly suggests the existence of different control mechanisms for surface motility in these two strains [25]. A transcriptomic analysis of a *fadD* mutant of *S. meliloti* strain Rm1021 under swarming-inducing conditions showed that iron and also genes required for siderophore rhizobactin 1021 (Rhb1021) synthesis are critical for surface translocation of the wild-type strain Rm1021 [25,26]. *S. meliloti rhb* mutants that are unable to produce the siderophore are non-motile on the surface of semisolid media. On the other hand, an *rhtA* mutant, which lacks the outer membrane receptor for Rhb1021 utilization, is motile indicating that the swarming deficiency shown by *rhb* mutants was not due to iron deficiency and furthermore, that Rhb1021’s involvement in swarming was exerted outside the cell. Surfactant properties inherent to the Rhb1021 structure, a citrate-based siderophore containing a long-chain fatty acid, could be responsible for the promotion of surface translocation in *S. meliloti*. Interestingly, the lack of a functional *fadD* gene restored surface motility in Rhb1021-deficient strains, indicating that the effect caused on surface motility by *fadD* loss-of-function is epistatic to mutations affecting siderophore production. Also, the same study showed that high iron conditions inhibited swarming motility in Rm1021, most likely by preventing Rhb1021 production. This inhibitory effect, however, was not observed in mutants lacking either RirA, an iron limitation response regulator, or FadD [25]. The *rirA* mutant’s phenotype could be explained by the bacteria’s ability to produce Rhb1021 under high iron conditions. However, the mechanism responsible for the iron-independent swarming phenotype shown by *fadD* mutants is unknown.

The connection between swarming motility and biofilm formation in *S. meliloti* has not yet been explored. In this work, we investigated whether factors known to influence swarming motility in *S. meliloti* have an impact on its capability to form biofilms. The study was performed using ExpR-deficient strains that display different surface motility behaviors. Although *expR*^−^ mutants are reported not to form the type of highly structured biofilms observed for *expR*^+^ strains, our findings show that, similar to flagella, Rhb1021 is required for both surface translocation and biofilm formation. In addition, data obtained from this study provide evidence for the existence in *S. meliloti* of common regulatory mechanisms governing surface motility and biofilm formation in which iron, the iron global regulator RirA, and FadD have important roles.

## Results

### Biofilm formation analysis in *S. meliloti* Rm1021 and GR4 strains

The existing knowledge on swarming motility in *S. meliloti* has mostly been obtained from studying the ExpR-deficient reference strains Rm1021 and GR4. In this way, the interference of a sliding motility promoted by EPS II production can be avoided [24-26]. Rm1021 and GR4 strains show different surface motility phenotypes. Rm1021 spreads over semisolid surfaces using flagella-dependent and -independent mechanisms, whereas GR4 is non-motile under the same conditions [25,26]. We thus tested whether this difference in behavior on semisolid surfaces could correlate with distinct biofilm formation abilities by analyzing biofilms established by these two strains on both abiotic (PVC and glass) and biotic surfaces (alfalfa roots).

Swarming motility, like biofilm formation, is highly influenced by medium composition [8,17,18,20,24]. In ExpR-deficient *S. meliloti* strains, swarming motility has only been shown to occur on semisolid MM [24-26]. This medium contains a relatively high phosphate concentration (3.5 mM), a condition known to slightly increase biofilm formation in *expR* mutants compared to that observed under low phosphate conditions (0.1 mM) [18]. To rule out any influence of the medium composition and to be able to correlate the swarming behavior with the biofilm formation ability of the different strains, *in vitro* biofilm formation was analyzed by growing cells in MM as described in the Methods section.

Under our experimental conditions, all the different *S. meliloti* strains used in this study exhibited poor biofilm formation capabilities on PVC microtiter plates, making it difficult to discern any significant differences among treatments (data not shown). Therefore, we restricted our biofilm formation experiments to glass and plant surfaces.

**Figure 1:**
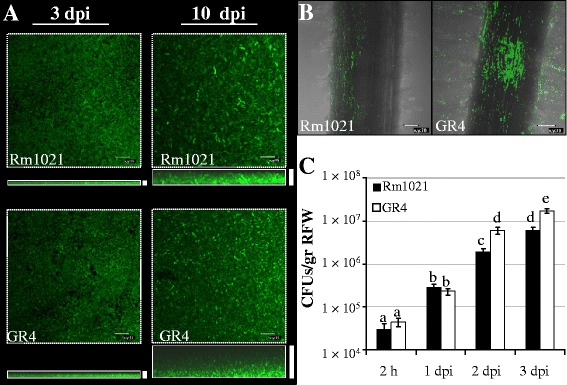
**Biofilm formation of** ***S. meliloti*** **ExpR defective strains Rm1021 and GR4. A)** Confocal images showing the *xy* and *xz* planes of biofilm development on chambered cover glass slides at 3 and 10 dpi. A thick, white bar placed next to the corresponding xz plane represents the thickness of each biofilm. Bars, 15 μm. **B)** Confocal images of alfalfa roots inoculated with GFP-labelled Rm1021 and GR4 strains at 3 dpi. Bars, 70 μm. **C)** Colony forming unit (CFU) counts recovered from alfalfa roots. Data are expressed per gram of root fresh weight (RFW). Error bars indicate the standard error from the mean. According to the Games-Howell test (*P* ≤ 0.05), values followed by the same letter do not differ significantly.

The ability to develop biofilms on glass surfaces was examined by growing green fluorescent protein (GFP)*-*labelled *S. meliloti expR* mutant strains on chambered covered glass slides. Confocal imaging of GFP-labelled GR4 and Rm1021 strains was performed during a 10-day-long experiment to observe the three-dimensional structure of the biofilms developed by these two strains. By 3 days post-inoculation (dpi), both Rm1021 and GR4 developed the unstructured, flat biofilms characteristic of static cultures of *expR* mutant strains (Figure 1A) [18]. Biofilm thickness increased with time for the two *S. meliloti* strains, reaching the maximum at the end of the experiment (10 dpi) (viewed on the xz planes, Figure 1A). At this final stage, biofilms developed by GR4 were clearly thicker than those formed by Rm1021 (compare z axes in Figure 1A), although GR4 cell density within the biofilm seemed to decrease with height. These differences in biofilm thickness seem to be specific for biofilm development because both Rm1021 and GR4 exhibited similar growth rates in liquid MM (data not shown).

As a complement to the experiments on abiotic surfaces, biofilm formation on alfalfa roots was examined. Three dpi, the root surfaces inoculated with either Rm1021 or GR4 were covered with microcolonies that remained attached along the oldest part of the root even after extensive washing (Figure 1B). Very few cells adhered to the younger root tissues and apical region. However, it is worth noting that more cell clusters were observed colonizing the root surface and especially the root hair zone of plants, when inoculated with GR4 (Figure 1B). Although the number of CFUs per gram of root tissue revealed no differences in the attachment ability of Rm1021 and GR4 2 h and 24 h post-inoculation, the number of GR4 cells attached to root surfaces 2 and 3 dpi was approximately 3-fold higher than the values for Rm1021 (Figure 1C). Overall, this indicates that GR4 is more efficient than Rm1021 in establishing biofilms on glass and root surfaces.

### Iron controls biofilm development in *S. meliloti*

**Figure 2:**
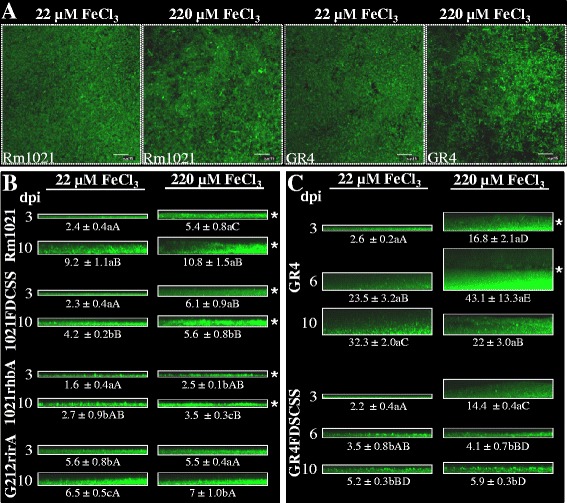
**Biofilm formation of** ***S. meliloti*** **strains on glass surfaces in response to iron availability. A)** Confocal Laser Scanning Microscopy (CLSM) images showing the architecture of 3-day-old biofilms developed by GFP-labelled Rm1021 and GR4 cells on chambered cover glass slides after growth in MM containing different concentrations of FeCl_3_. Bars, 15 μm. **B)** CLSM images showing the thickness (represented by the *xz* plane) of 3- and 10-day-old biofilms developed by Rm1021 and its derivative mutant strains on chambered cover glass slides in response to low (22 μM FeCl_3_) and high (220 μM FeCl_3_) iron availability. **C)** CLSM images showing the thickness (represented by the *xz* plane) of 3-, 6-, and 10-day-old biofilms developed by GR4 and GR4FDCSS. The mean thickness (± SD) of biofilms formed by the different strains under the two iron conditions is shown in parenthesis below the representative CLSM image. Values having different letters are significantly different from each other (Tukey’s test, *P* < 0.05). Lowercase letters indicate statistical differences between the thickness of the wild type strain and its derivative mutant biofilms developed under the same conditions. Capital letters are used to indicate the statistical differences observed in the biofilms developed under the different conditions by each strain. Structured biofilms developed in MM containing 220 μM of FeCl_3_ are indicated with an asterisk (*). Each experiment was repeated three times.

Iron regulates biofilm formation in multiple bacterial species. In some species, iron limitation induces biofilm development [28,29] whereas in others, biofilm inhibition occurs in response to low iron availability [30-32]. Previous studies from our group showed that high concentrations of iron inhibit surface motility in *S. meliloti* reference strain Rm1021 [25]. To investigate whether iron could also affect the development of surface-associated communities in the two *S. meliloti* strains, we compared the biofilms formed by the ExpR defective strains GR4 and Rm1021 on glass under high iron conditions (MM containing 220 μM FeCl_3_, herein referred to as MMh; i.e. iron-rich conditions that block siderophore production in Rm1021 [25]), with those formed under low iron levels (22 μM FeCl_3_, referred to as MM. The latter conditions allow siderophore production in Rm1021 [25]). The presence of high iron did not lead to any significant effect on the growth curves shown by liquid cultures of Rm1021 and GR4 (data not shown). In contrast, analysis of biofilms established on glass revealed significant changes in the biofilms formed by the two strains of *S. meliloti* in iron-supplemented MM. Growth of both Rm1021 and GR4 in the presence of 220 μM FeCl_3_ induced the formation of sponge-like structured biofilms distinguishable from the flat biofilms established in low iron conditions (Figure 2A). Moreover, iron addition to the medium significantly increased the thickness of biofilms developed by the two reference strains at early stages (3 dpi) (Figure 2B, C), with this effect being especially obvious in the GR4 genetic background. Ten days after inoculation, no noticeable differences in thickness could be observed between biofilms developed by Rm1021 under low and high iron conditions (Figure 2B), although they exhibited the same differences in overall structure as observed in biofilms 3 dpi; i.e. flat and uniform layers of cells under low-iron conditions *versus* sponge-like structured biofilms in iron-rich medium (indicated with an asterisk in Figure 2B; see more detail in Additional file 1). The thickest and most structured biofilms were observed for strain GR4 after 6 days of growth in iron-replete medium (Figure 2C; Additional file 2). Later, at 10 dpi, the biofilm developed by GR4 under these conditions became thinner and lost the characteristic sponge-like structure, suggesting the initiation of biofilm dispersal. These results indicate that high iron availability enhances biofilm formation in *expR* mutant strains of *S. meliloti*, triggering the formation of more structured and thicker biofilms than those formed under low iron conditions. These effects are more pronounced in GR4 than in Rm1021, suggesting that iron-regulated mechanisms governing biofilm development differ in these two strains.

### *S. meliloti* mutants altered in surface motility are impaired in biofilm development on glass surfaces

Because *S. meliloti fadD*, *rhb* and *rirA* null mutants have been reported to show an altered motility phenotype compared to their corresponding parental strain in surface motility assays, we examined biofilm formation ability in these mutants in an attempt to identify candidate genes that could be involved in both surface motility and biofilm formation.

We previously showed, that in addition to its function in lipid metabolism, *fadD* is involved in the control of surface motility in *S. meliloti* [24,25,33]. By yet unknown mechanisms, a *fadD* mutation: i) promotes surface migration in ExpR-defective reference strains Rm1021 and GR4; ii) is epistatic to *rhb* mutations because it enables surface translocation of otherwise non-motile siderophore Rhb1021-defective mutants; and iii) relieves the inhibition of surface motility caused by high iron conditions. Our next goal was to determine whether *fadD* could also contribute to biofilm formation in *S. meliloti*. Growth rates of Rm1021 and GR4 derivative *fadD* mutants were similar to the rates of their corresponding parent strains, in both MM and MMh. However, confocal images of biofilms established on glass surfaces revealed that irrespective of iron levels, the *fadD* mutation negatively affected biofilm development in both strains Rm1021 and GR4 (Figure 2 B, C; Additional files 1 and 2). Under both low and high iron conditions, the thickness of *fadD* mutant biofilms was significantly reduced at later stages of biofilm development compared to the corresponding parent strain (10 dpi, Figure 2 B, C). In addition, *fadD* loss-of-function in the GR4 genetic background also prevented the formation of highly structured biofilms under high iron conditions (Figure 2C; Additional file 2). These results indicate that the fatty-acid metabolism-related enzyme FadD participates by a yet unknown mechanism in normal biofilm development in *S. meliloti*.

Two other *S. meliloti* mutants affected in surface translocation and assessed for biofilm development in this study are *rhb*, which is affected in iron uptake and *rirA* an iron homeostasis mutant. Rm1021 *rhb* mutants are unable to synthesize the siderophore Rhb1021 and cannot translocate on semisolid surfaces. This, together with the demonstration that the siderophore uptake *rhtA* mutant exhibits surface movement [25], suggests a specific role for the siderophore in Rm1021 surface motility in addition to its function in iron nutrition. In contrast to Rm1021, GR4 does not produce Rhb1021, which might explain the inability of this strain to translocate over semisolid MM [24,25].

Analyses of biofilm formation on glass surfaces made it possible to see that the Rm1021 *rhbA* mutant is highly impaired in biofilm development. Although no significant changes were detected in the structure exhibited by the *rhbA* mutant biofilms compared to that shown by Rm1021 biofilms in either low or high iron conditions, significant reductions in mutant biofilm thickness were observed at later stages (10 dpi) compared to wild-type Rm1021 (Figure 2B; Additional file 1). Similar results were obtained with an in-frame *rhbD* deletion mutant (data not shown; [34]). No significant changes in growth rates have been detected in Rm1021 *rhb* mutants compared to the wild-type strain in liquid MM or MMh (data not shown). More importantly, the fact that increased iron availability conditions, in which Rhb1021 production is abolished [25], could not restore biofilm development of the *rhbA* mutant suggests that, Rhb1021 synthesis is essential at some stages of biofilm development and is involved in a way that is not exclusively related to its function as siderophore.

On the other hand, we demonstrated that loss-of-function of the iron response regulator RirA in Rm1021 does not affect surface motility under low iron conditions, but leads to a hypermobile phenotype in the presence of high levels of iron, suggesting a role for this regulator in the control of surface motility in response to iron concentration [25]. Under iron-sufficient conditions, *S. meliloti rirA* mutants cannot down-regulate iron uptake systems, including siderophore Rhb1021 synthesis, which leads to oxidative stress and a corresponding reduction in growth [35].

Confocal Laser Scanning Microscopy (CLSM) images of biofilms established on glass surfaces by the *rirA* mutant revealed that the mutation affected normal biofilm development (Figure 2B; Additional file 1). Under low iron conditions, the *rirA* mutant formed flat, unstructured biofilms, which at early stages (3 dpi) were thicker than those developed by its parental strain Rm1021. However, *rirA* mutant biofilms did not increase in thickness with time; this led to the formation of slightly thinner biofilms than the ones formed by Rm1021 at the end of the experiment. The most relevant difference between Rm1021 and *rirA* mutant biofilms was observed under high iron conditions. RirA loss-of-function abolished the formation of thicker and sponge-like structured biofilms, and thus no changes were detected in *rirA* mutant biofilms formed in iron-replete medium compared to those formed under low iron levels (Figure 2B; Additional file 1). These results strongly suggest that this regulator plays a pivotal role in controlling functions required for biofilm development in response to iron availability.

### *S. meliloti fadD*, *rhb* and *rirA* mutants show defects in alfalfa root colonization

**Figure 3:**
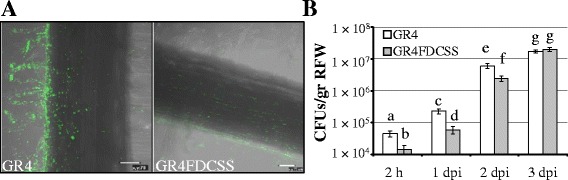
***In planta*** **biofilm formation by GR4 and its** ***fadD*** **derivative mutant. A)** Confocal images of biofilms established on alfalfa root surfaces 3 dpi. Bars, 70 μm. **B)** Colony forming unit (CFU) counts recovered from alfalfa roots. Data are expressed per gram of root fresh weight (RFW). Error bars indicate the standard error from the mean of three independent experiments. According to the Games-Howell test (*P* ≤ 0.05) values followed by the same letter do not differ significantly.

**Figure 4:**
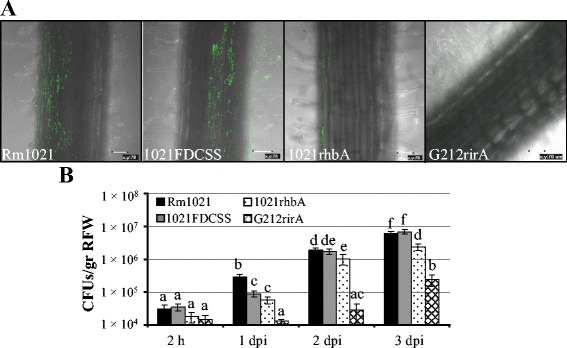
***In planta*** **biofilm formation by Rm1021 and** ***fadD*** **,** ***rhbA,*** **and** ***rirA*** **mutants. A)** Confocal images of biofilms established on alfalfa root surfaces 3 dpi. Bars, 70 μm. **B)** Colony forming unit (CFU) counts recovered from alfalfa roots. Data are expressed per gram of root fresh weight (RFW). Error bars indicate the standard error from the mean of three independent experiments. According to the Games-Howell test (*P* ≤ 0.05) values followed by the same letter do not differ significantly.

We decided to investigate whether mutations in *S. meliloti* causing alterations in two different surface-associated phenotypes (swarming and biofilm formation) could also have an impact on biofilm development *in planta,* i.e. alfalfa root colonization. Confocal images and CFU counting revealed that the GR4-derivative *fadD* mutant (GR4FDCSS) exhibited a significantly decreased ability to attach to and to colonize root surfaces compared to GR4 (Figure 3), although this difference diminished with time (see 3 dpi in Figure 3B). Reduced biofilm formation on alfalfa roots was also detected for 1021FDCSS (Figure 4), but the effect was transitory and less noticeable than for GR4FDCSS. We could discern differences only when counting CFU 1 dpi, at which time the number of attached 1021FDCSS cells was approximately 3-fold lower than that of Rm1021 (Figure 4B). No significant differences in root colonization were detected at later stages by either confocal imaging or CFU counting between 1021FDCSS and Rm1021 (Figure 4 A, B). Therefore, a *fadD* mutation in *S. meliloti* negatively interferes with normal biofilm development on root surfaces, although the effects are more pronounced in the GR4 strain background than in the Rm1021 genetic background.

The Rm1021 siderophore defective *rhbA* mutant was also impaired in establishing bacterial communities on the surface of alfalfa roots (Figure 4A). In contrast to the significant root colonization observed for Rm1021, plant roots inoculated with the *rhbA* mutant supported only small groups of a few bacteria that were attached to the central part of the root and root hairs. CFU counting of root-associated bacteria confirmed this result and demonstrated that the number of *rhbA* mutant cells colonizing alfalfa roots was 5, 2, and 2.6-fold lower that those observed for Rm1021 at 1, 2, and 3 dpi, respectively (Figure 4B).

The most striking phenotype was observed for the *rirA* mutant, which was severely impaired in alfalfa root colonization. Only a few mutant cells could be observed to be attached to the main root and root hairs by CLSM (Figure 4A). Moreover, CFU counting revealed that the number of *rirA* mutant cells recovered from alfalfa roots was 22, 68, and 25-fold lower than for Rm1021 at 1, 2, and 3 dpi, respectively (Figure 4B). Thus, the iron homeostasis-related genes *rhb* and *rirA* are critical in strain Rm1021 for biofilm development on glass and alfalfa root surfaces.

## Discussion

**Figure 5:**
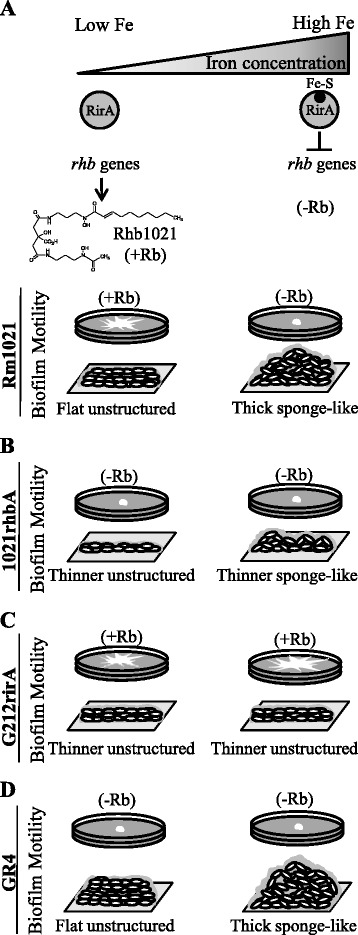
**Effects caused by iron in surface motility and biofilm formation in different** ***S. meliloti*** **strains. A)** Surface motility and development of structured biofilms are inversely regulated by iron levels in Rm1021. Under low-iron conditions, the iron regulator RirA is free and its repressor activity is low allowing high expression of *rhb* genes and the corresponding production of siderophore Rhb1021 (+Rb) in the reference strain Rm1021. Amphiphilic properties associated with Rhb1021 facilitate surface spreading and the formation of flat, unstructured biofilms. Under iron-replete conditions, the number of RirA molecules bound to Fe-S clusters rises leading to the repression of *rhb* genes and as a result, Rhb1021 production is abolished (−Rb), thereby preventing surface motility. This together with putative additional functions regulated by iron favors biofilm formation. Most likely, even under iron-replete conditions, some areas within a wild type biofilm might suffer low iron availability, which could trigger Rhb1021 production and impact biofilm architecture. **B)** The inability to produce Rhb1021 caused by *rhb* loss-of-function abolishes surface motility and reduces biofilm thickness regardless of iron levels in the medium. **C)** Uncontrolled Rhb1021 synthesis in the *rirA* mutant allows surface translocation under iron-replete conditions but prevents the formation of structured mature biofilms. **D)** Unlike Rm1021, the wild type strain GR4 does not produce Rhb1021 and does not show surface translocation under low-iron conditions. However, like in Rm1021, iron-replete conditions promote the formation of structured and thick biofilms.

**Figure 6:**
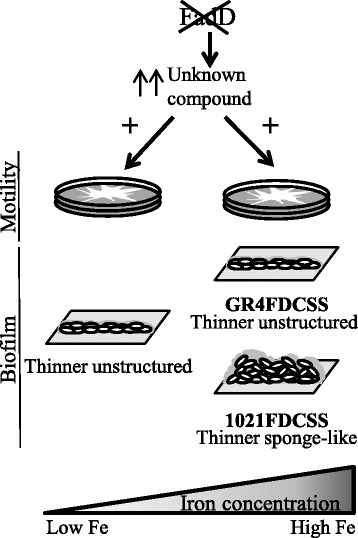
**Effects of** ***fadD*** **loss-of-function in surface motility and biofilm formation by Rm1021 and GR4.** *fadD* knock-out mutation could lead to the accumulation of as yet an uncharacterized fatty acid-related compound whose synthesis is not under iron control. This compound promotes surface motility of both Rm1021 and GR4 even in the absence of Rhb1021 and thus negatively interferes with biofilm development. Impairment in biofilm formation caused by *fadD* mutation is more drastic in the GR4 strain in that it affects not only the thickness of biofilms, but also abolishes the development of the characteristic sponge-like structure of biofilms formed under iron-replete conditions. Comparisons of biofilms are made relative to the corresponding parent strain.

Numerous investigations on swarming motility and biofilm formation in pathogenic bacteria have demonstrated the intimate link between these two multicellular surface-associated behaviors as well as their impact on host colonization and infection. In contrast, very little is known about genes and regulatory mechanisms governing these two opposite lifestyles in *Rhizobium*. The aim of this work was to investigate the existence of a possible connection between swarming motility and biofilm formation in *Sinorhizobium meliloti* by analyzing the effect on biofilm development in different genetic backgrounds and under environmental conditions known to influence surface motility in the alfalfa symbiont. Our study was performed using two ExpR-deficient strains that display different surface motility behaviors [24-26]. Although these strains cannot produce EPS II, the matrix for the development of highly structured biofilms reported for *expR*^+^ strains [18], our results strongly support the existence of a link between surface motility and biofilm formation in *S. meliloti*. Moreover, we show that the siderophore Rhizobactin 1021 (Rhb1021) is important for biofilm formation, adding to its previously reported involvement in surface motility and iron nutrition. Thus, Rhb1021 represents a third component together with flagella and EPS II, which are known to be essential for the two surface-associated phenotypes. In addition, we provide evidence for an inverse co-regulation of surface motility and biofilm formation in which iron is an important environmental cue, whereas *fadD* and *rirA* are genetic determinants that influence both surface-associated phenotypes by independent, but as yet unknown, mechanisms. Figures 5 and 6 summarize the results that support these conclusions and illustrate models explaining the involvement of iron, RirA, and the siderophore Rhb1021. In addition, a FadD-related compound is proposed to be involved in the control of surface motility and biofilm formation in *S. meliloti*.

The part that siderophore Rhb1021 in *S. meliloti* plays in biofilm development is indicated from the phenotype shown by *rhb* mutants on glass surfaces. Regardless of iron levels, these mutants formed thinner biofilms than the parent strain (Figure 2B; Figure 5 A and 5B). The mechanism(s) by which Rhb1021 contributes to biofilm formation in *S. meliloti* have not been investigated, but in other bacteria, siderophores are implicated in biofilm development. The mechanisms whereby most of these siderophores act in establishing surface-associated bacterial communities are related to the important regulatory function that intracellular iron plays in biofilm formation in different bacteria. For example, in *Pseudomonas aeruginosa,* the pyoverdine system is required for normal biofilm development under iron-depleted conditions, but high iron concentrations suppress the mutant defect [30]. More recently, enterobactin was shown to be required for biofilm development in reduced-genome *Escherichia coli* [36]. In contrast to *P. aeruginosa*, biofilm development by the *S. meliloti* Rhb1021-defective mutant is not restored under high iron conditions (Figure 2B; Figure 5 A and 5B), strongly suggesting that Rhb1021’s involvement in biofilm establishment may not be exclusively related to iron acquisition. Rhb1021 is a citrate-based dihydroxamate siderophore with an asymmetric structure conferred by the presence of a long-chain fatty acid ((E)-2-decenoic acid) that gives Rhb1021 amphiphilic properties [37]. We propose that the function exerted by this siderophore in surface motility and biofilm formation is likely to be linked to its surfactant activity. Different molecules with surfactant activities that are involved in swarming motility such as lipopeptides or rhamnolipids produced by *P. aeruginosa*, have been shown to influence bacterial biofilms by having different functions at different stages of biofilm development [38-42]. Another non-excluding possibility is that Rhb1021 contributes to surface-associated phenotypes by acting as a signal molecule, a function that has been reported for other siderophores and surfactants [43,44]. In either case, the production of Rhb1021 within the biofilm must be tightly regulated to allow for normal biofilm development because exogenous application of purified siderophore to the *rhb* mutant not only does not rescue biofilm formation, but also significantly decreases biofilm formation in the parent strain (data not shown, [34]). Further investigations will be necessary to elucidate the mechanism(s) of action of Rhb1021 in biofilm formation.

Several results obtained during this study suggest that, as in other bacteria, an inverse co-regulation exists between the ability to translocate over surfaces and biofilm formation in *S. meliloti*. Strain GR4, which is less prone to translocate over surfaces under laboratory conditions than strain Rm1021, establishes biofilms on abiotic and root surfaces more efficiently. The difference is especially noticeable under high-iron conditions where GR4 develops a very thick and structured biofilm. Thus, similar to the control of swarming motility, different regulatory mechanisms govern biofilm development in these two ExpR defective strains. GR4’s increased biofilm formation efficiency could also explain its ability to elicit more efficient nodule formation compared to strain Rm1021 [45].

Furthermore, we found that high-iron conditions that inhibit surface motility in *S. meliloti* induce biofilm formation (Figure 2 and Figure 5) demonstrating that iron inversely co-regulates the two surface-associated phenotypes. Iron has been shown to regulate biofilm formation in multiple bacterial species. In some, such as *Legionella pneumophila* and *Staphylococcus aureus*, iron limitation induces biofilm formation [28,46], whereas in other species, including *E. coli, P. aeruginosa*, and *V. cholerae,* iron limitation inhibits biofilm formation [30,32,47-49]. These different responses to iron availability might be the result of differences in iron acquisition systems, characteristics of the cell surface, or different properties of the extracellular matrix. The involvement of iron in *S. meliloti* biofilm formation shares some similarities with its role in *P. aeruginosa* biofilm development. Both bacteria form flat unstructured biofilms under low iron conditions. In *P. aeruginosa*, this phenotype was explained by iron-regulated production of rhamnolipids [50]. It is tempting to speculate that Rhb1021, with surfactant properties whose production is also controlled in response to iron levels, might also be responsible for the effects caused by iron in *S. meliloti* biofilm structure. With the data available so far, it is difficult to know what is the biological role in *S. meliloti* (if there is any) of the inverse co-regulation of surface motility and biofilm formation in response to iron availability. A possibility is that *S. meliloti* uses iron-sensing as a signal that controls the transition from the free-living lifestyle in soil to that of bacteria in symbiosis with its plant host. Although iron is abundant, its bioavailability in soils is very low due to its low solubility under aerobic conditions, especially in neutral or alkaline soils [51]. In neutral soils, the concentration of free Fe^3+^ is at best 10^−17^ mol/L. These poor iron conditions would favor bacterial motility over sessility. However, the situation in the rhizosphere might be different as the result of plant activities aimed to acquire iron. Thus, the activity of a root ferric reductase protein that reduces Fe (III) to Fe (II), and acidification of the root environment to improve Fe solubilization and the efficiency of the ferric reductase protein [52] could increase the concentration of free iron available for rhizosphere-dwelling microorganisms such as *S. meliloti*, thereby favoring biofilm formation and an intimate association with the plant host.

Lastly, *fadD* and *rirA* null mutations interfere with *S. meliloti* surface translocation control by promoting motility under non-permissive conditions. The finding that these mutations also impede the development of mature biofilms on glass surfaces (Figure 2, 5, 6) is convincing evidence for the inverse co-regulation of surface motility and biofilm formation in this *Rhizobium* species. The likely independent mechanism(s) by which these two genes participate in such control are still unknown and is the subject of current investigations. The inability of the *rirA* mutant to develop the thick and structured biofilms observed for the wild-type strain under high iron conditions, could be simply the result of growth defects caused by oxidative stress due to derepressed iron uptake. In addition, the increased and deregulated production of Rhb1021 associated with the *rirA* mutation inhibits biofilm formation in *S. meliloti* just as overproduction and deregulation of rhamnolipids impede biofilm formation in *P. aeruginosa* [40]. Our data do not exclude the possibility that, besides Rhb1021 production, RirA also regulates other functions important for biofilm development.

Our previous work revealed that *fadD* loss-of-function deregulates normal surface motility in *S. meliloti*. Here we show that *fadD* loss-of-function also obstructs normal biofilm development on both glass and root surfaces in the two *S. meliloti* strains studied. However, the effects are more pronounced in the GR4 strain than in the Rm1021 genetic background. In contrast to the *rirA* mutant, the defects in biofilm formation shown by the two different *fadD* mutants are not due to differences in growth or Rhb1021 production relative to the wild type suggesting the involvement of a different mechanism [25]. Interestingly, gene expression analyses indicate that *fadD* transcription is induced under low iron levels in both Rm1021 and GR4 (Additional file 3). Whether this effect is somehow relevant to iron control over surface motility and biofilm formation in the wild-type situation is unknown, but if this is the case, it would be lost in *fadD* loss-of-function mutants. In *S. meliloti,* FadD allows the utilization of exogenous and endogenous long chain fatty acids via their activation with CoA [33]. In culture, *S. meliloti fadD* mutants accumulate a mixture of free fatty acids during stationary phase [33], and fatty acids and fatty acid-related signals are known to influence surface motility and biofilm formation in different bacteria (reviewed in [53-55]). The hypothesis of surface-associated phenotypes in *S. meliloti* being affected either by changes in cellular fatty acid composition and membrane fluidity or by the accumulation of fatty acid-related compounds acting as biosurfactants and/or signal molecules deserves further investigation.

Although it is accepted that biofilm formation allows rhizobia to survive under adverse environmental conditions, the exact function of biofilms in the *Rhizobium*-legume symbiosis remains elusive [20]. Recently, studies of a *nodD1* mutant and a quorum sensing-defective strain demonstrated that biofilm formation is crucial for optimal root colonization and symbiosis between *Sinorhizobium fredii* and soybean plants [56]. Our data also support the importance of biofilm formation in promoting an effective symbiosis with the legume host. Although able to initiate nitrogen-fixing nodules, the strains used in this study had been earlier described as being less competitive in nodule formation, and here were found to be less efficient in biofilm formation. This is true for Rm1021 *vs* GR4 [45] and the *fadD* mutant *vs* its parental strain [24]. Likewise, *rhb* and *rirA* mutants of *S. meliloti* are able to nodulate and fix nitrogen in symbiosis with alfalfa plants [57,58]. However, by performing nodule competition experiments against the corresponding parental strain, we found that the *rhb* mutant was as competitive as the parental strain (complementation caused by wild-type siderophore production could hinder any symbiotic defect), but a significant reduction in competitive ability was observed for the *rirA* mutant, which occupied 27% fewer nodules than the wild-type strain (data not shown). It is conceivable that biofilm formation defects in rhizobial strains might cause only subtle symbiotic deficiencies, which occur at the beginning of the interaction during the root colonization process. To be an efficient root colonizer would be an important attribute under field conditions where competition with other microorganisms and adverse environmental conditions impose a strong selective pressure.

Moreover, the fact that *fadD* and *rirA* mutants are affected in surface motility, biofilm formation, and plant root colonization strongly suggests that genes that participate in the coordinated control of surface-associated phenotypes are required for optimal interaction with the host plant. Future research on iron acquisition systems and iron-dependent regulation in *S. meliloti* as well as of the effects caused by the disruption of *fadD* will undoubtedly facilitate the identification of new components that govern biofilm formation and plant colonization in this group of bacteria.

## Conclusions

In recent years, a growing appreciation has been developing of the impact that biofilm formation has on the success and outcome of plant-microbe interactions. Bacterial biofilms seem not only to foster bacterial survival against environmental stresses, but also to promote the initiation of beneficial plant-microbe associations by maintaining a dense population of cells in a specific location for a sufficient length of time. Our research demonstrates that by investigating the molecular mechanisms involved in bacterial surface motility, we can uncover components that are key factors for biofilm formation and also essential for plant root colonization. The major molecular mechanisms known to govern surface motility in *S. meliloti* were found to be either essential for biofilm formation or to regulate both processes inversely in this rhizobial strain. We also conclude that the function exhibited by rhizobactin 1021 in surface motility and biofilm development goes beyond its role as siderophore. Although Rhb1021 is not essential for the formation of nitrogen-fixing nodules, we demonstrate that it contributes to optimal root colonization, a characteristic that might be relevant under field conditions. Furthermore, the environmental concentration of iron and genes *fadD* and *rirA* were shown to coordinate surface motility and biofilm formation inversely. This study supports the hypothesis that the intimate regulation of surface motility and biofilm formation enables rhizobia to colonize roots optimally and to initiate the establishment of the symbiosis with alfalfa plants successfully.

## Methods

### Bacterial strains, plasmids, and growth conditions

The bacterial strains and plasmids used in this study are listed in Table 1. *E. coli* strains were grown in Luria-Bertani medium (LB) [59] at 37°C. *S. meliloti* strains were routinely cultured in Tryptone-Yeast extract complex medium (TY) [60], or in Minimal Medium (MM) containing glutamate (6.5 mM), mannitol (55 mM), mineral salts (K_2_HPO_4_, 1.3 mM; KH_2_PO_4_^.^3H_2_O, 2.2 mM; MgSO_4_^.^7H_2_O, 0.6 mM; CaCl_2_^.^2H_2_O, 0.34 mM; FeCl_3_^.^6H_2_O, 0.022 mM; NaCl, 0.86 mM) and vitamins (biotin (0.2 mg/L); calcium pantothenate (0.1 mg/L)) [61] at 30°C. Iron-replete MM (MMh) containing 220 μM of FeCl_3_ was prepared by adding the appropriate volume of a 100-fold concentrated stock solution of FeCl_3_ to MM without iron. The plasmid pHC60 [62] was introduced into *S. meliloti* strains by a biparental mating using the *E. coli* mobilizing strain S17-1. Antibiotics were added, as appropriate, at the following final concentrations: for *E. coli*, tetracycline (Tc) 10 μg/ml; for *S. meliloti,* streptomycin (Sm) 200 μg/ml, spectinomycin (Sp) 100 μg/ml, kanamycin (Km) 100 μg/ml, neomycin sulphate (Nm) 100 μg/ml, and Tc 10 μg/ml. The *fadD* mutant strain GR4FDCSS was obtained following the same procedure described for 1021FDCS [25].

**Table 1:** **Bacterial strains and plasmids used in this study**

**Strain or plasmid**	**Relevant characteristics** ^**a**^	**Source or reference**
*Escherichia coli*		
S17.1	*thi*, *pro*, *recA*, *hsdR*, *hsdM*, Rp4Tc::Mu, Km::Tn*7*; Tp^r^, Sm^r^, Sp^r^	[63]
*Sinorhizobium meliloti*		
GR4	Wild-type	[64]
GR4FDCSS	GR4 (Δ*fadD*::SmSp), Sm^r^ Sp^r^	This study
Rm1021	SU47 *expR102*::IS*Rm*2011-1, Sm^r^	[65]
1021FDCSS	Rm1021 (Δ*fadD*::SmSp), Sm^r^ Sp^r^	[25]
1021rhbA	Rm1021 *rhbA*::Tn*5lac*; Sm^r^ Nm^r^	[26]
G212rirA	Rm1021 (lac^−^, *rirA*::Km), Sm^r^ Km^r^	O’Connell, M.
Plasmids		
pHC60	IncP plasmid constitutively expressing GFP, Tc^r^	[62]

^a^Tp^r^, Sm^r^, Sp^r^, Nm^r^, Km^r^, Tc^r^: abbreviations for resistance to trimethoprim, streptomycin, spectinomycin, neomycin, kanamycin, and tetracycline.

### Biofilm formation observation by confocal laser scanning microscopy (CLSM)

A confocal laser scanning inverted microscope Nikon Eclipse TE2000-U (Nikon Instruments, Melville, NY, USA) was employed to visualize the structure and different stages of biofilm formation of rhizobial cells, which constitutively expressed green fluorescent protein (GFP). Biofilms were established on chambered cover glass slides containing a 1-μm-thick borosilicate glass base (Nunc Lab-Tek no. 155411, THERMO Fisher Scientific, Waltham, Massachusetts, USA) as described [66]. The GFP-labelled rhizobial cells were grown to OD_590_ = 1.5-2.0 in MM. They were then washed twice with MM without iron and resuspended in MM containing the appropriate FeCl_3_ concentration (22 μM for low iron or 220 μM for iron-replete conditions) to OD_590_ = 0.2. The 1021rhbA-*gfp* strain, which could not reach a high OD in MM broth, was prepared by diluting a cell mass freshly grown on MM plates in MM broth. Five hundred microliters of the diluted cultures (OD_590_ = 0.2) were inoculated into each chamber and grown under static conditions at 30°C for up to 10 days. To prevent desiccation, the chambers were incubated in a sterile Petri dish under humidified conditions. At defined times, the medium was removed, and unbound bacteria were eliminated by washing each chamber with 500 μl of sterile water. Images of the biofilm were acquired by scanning with settings optimal for GFP (488-nm excitation with argon laser line and 500-nm long-pass emission) and were processed using the digital image processing Nikon software, EZ-C1 Freeviewer. Biofilm thickness values were calculated considering the number of CLSM sections on the z-axis and the distance between each section (0.2 μm).

### Plant assays

Alfalfa (*Medicago sativa* L.) seeds were sterilized and germinated as described by Olivares *et al.* [67]. One-day-old seedlings (1 cm long) were transferred to square Petri dishes containing nitrogen-free medium [67] solidified with 0.8% agarose (Pronadisa, Madrid, Spain) and overlaid with sterile filter paper moistened with 2 ml sterile water. To observe biofilm formation over the root surface, the seedlings were immediately inoculated with 30 μl of a GFP-labelled *S. meliloti* suspension containing approximately 1 × 10^6^ bacterial cells/ml. Cells for the rhizobial suspension were previously grown on TY plates at 30°C, and the cell mass was resuspended in TY broth up to an OD_600_ = 0.1 and further diluted 100-fold in sterile water to prepare an inoculum of approximately 3 × 10^4^ bacterial cells/plant. At pre-determined times, from one to four days, at least 10 roots inoculated with each rhizobial strain were extensively washed in sterile water, and placed on a microscope slide. The distribution of bacteria along the root was observed using a Leica DMI600B epifluorescence inverted microscope (Wetzlar, Germany). Digital images of biofilms on roots were taken using a Nikon Eclipse TE2000-U inverted microscope (488-nm argon laser excitation and 500-nm long-pass emission) and analyzed using digital image processing EZ-C1 Freeviewer Nikon software.

Colonization of the bacteria on the root and the root hairs was also evaluated by determining colony-forming units (CFUs). Two-day-old seedlings were inoculated with 30 μl of a suspension containing approximately 3 × 10^5^ bacterial cells, prepared as described above. At defined times, 15 seedlings were extensively washed with sterile water to remove the not-firmly attached bacteria, and any water excess was removed using sterile filter paper, and the roots were weighed. Three roots were placed into a 1.5 ml Eppendorf tube containing 1 ml of sterile TE buffer and the attached cells were released by 2 sonication pulses of 1 min each in an Ultrasons-H (J.P. Selecta, Barcelona, Spain) sonication bath with a pause time of 1 min between the pulses, and subsequently quantified by counting CFUs (normalized to grams of root). The experiment was repeated three times.

### Statistical analysis

Values presented are the means and standard deviation (SD) of repeated experiments. Data were subject to one-way ANOVA test followed by comparison of multiple treatment levels with the control using the Games-Howell test. Biofilm thickness data was analysed as counts of number of CLSM *z-*sections using generalized linear models (GLMs) followed by multiple comparisons of treatments with Tukey’s tests. Statistical analyses were performed with SPSS 15.0 and R version 3.1.0 for Windows (package “multcomp”).

## Additional files

Additional file 1:
**Effect of iron concentration and different gene mutations on the structure of Rm1021 biofilms.** Confocal laser scanning microscopy (CLSM) images showing the xy view of 3- and 10-day-old biofilms developed by GFP-labelled Rm1021 and Rm1021-derived *fadD*, *rhb* and *rirA* mutant cells on chambered cover glass slides after growth in MM containing different iron concentrations. All different strains develop flat unstructured biofilms when they are grown in MM containing low iron concentration (22 μM FeCl_3_). Growth in the presence of high iron conditions (220 μM FeCl_3_) induces the formation of sponge-like structured biofilms except in the case of the *rirA* mutant. Bars, 15 μm. Additional file 2:
**Effect of iron concentration and** ***fadD*** **-loss-of function on the structure of** ***S. meliloti*** **GR4 biofilms.** CLSM images showing the xy view of 3-, 6- and 10-day-old biofilms developed by GFP-labelled GR4 and *fadD* derivative mutant cells on chambered cover glass slides after growth in MM containing different iron concentrations. Both strains develop flat unstructured biofilms when they are grown in MM containing low iron concentration (22 μM FeCl_3_). In the presence of high iron conditions (220 μM FeCl_3_), the wild-type strain GR4 develops highly structured biofilms. The characteristic sponge-like structure of these biofilms is lost after 10 days, suggesting the initiation of biofilm dispersal. The *fadD* mutant (GR4FDCSS) is unable to develop structured biofilms even in the presence of iron-rich conditions. Bars, 15 μm. Additional file 3:
**Effect of iron concentration on** ***fadD*** **gene expression in** ***S. meliloti*** **GR4 and Rm1021.**
