# Supplementary figures and images for: Biofilm formation assessment in Sinorhizobium meliloti reveals interlinked control with surface motility
Source: BMC Microbiol. 2015 Mar 3;15:58. doi: 10.1186/s12866-015-0390-z (PMC4381460; doi:10.1186/s12866-015-0390-z)

## Slide 1
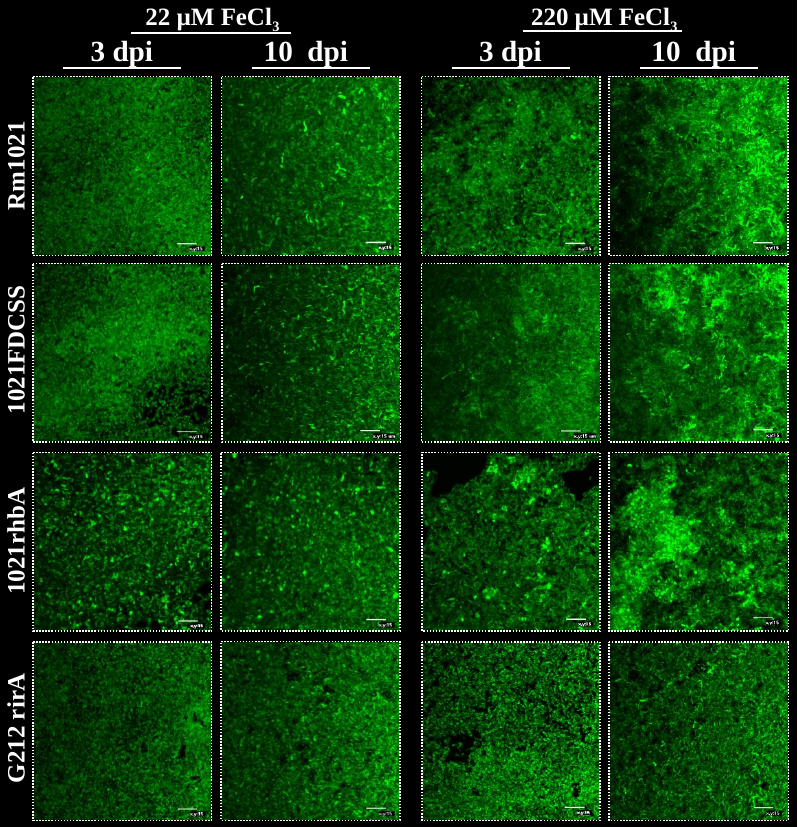

22 µM FeCl3
 220 µM FeCl3
3 dpi
10 dpi
3 dpi
10 dpi
 Rm1021
 1021FDCSS
 1021rhbA
 G212 rirA

Supplement: Additional file 1: — Effect of iron concentration and different gene mutations on the structure of Rm1021 biofilms. Confocal laser scanning microscopy (CLSM) images showing the xy view of 3- and 10-day-old biofilms developed by GFP-labelled Rm1021 and Rm1021-derived fadD, rhb and rirA mutant cells on chambered cover glass slides after growth in MM containing different iron concentrations. All different strains develop flat unstructured biofilms when they are grown in MM containing low iron concentration (22 μM FeCl3). Growth in the presence of high iron conditions (220 μM FeCl3) induces the formation of sponge-like structured biofilms except in the case of the rirA mutant. Bars, 15 μm. [file 12866_2015_390_MOESM1_ESM.pptx]

## Slide 1
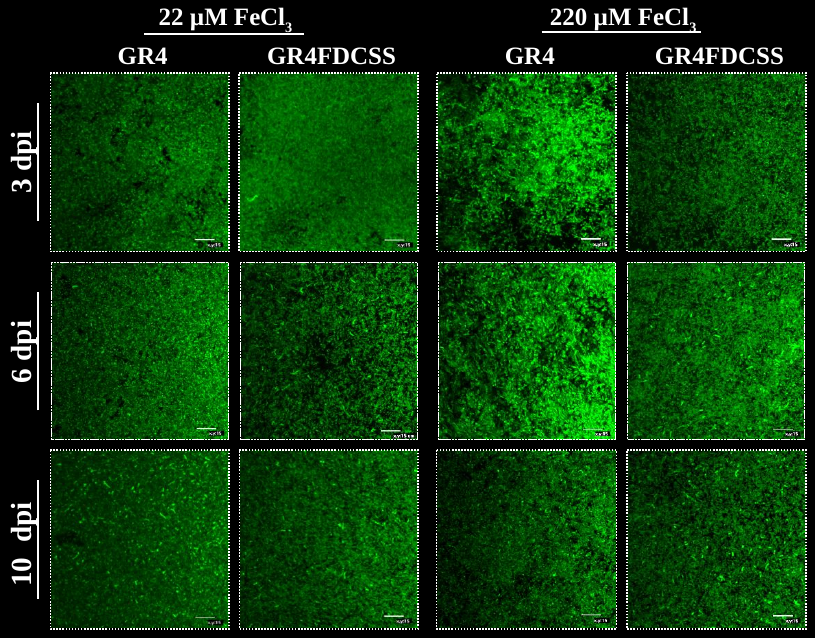

22 µM FeCl3
 220 µM FeCl3
 GR4
 GR4FDCSS
 GR4
 GR4FDCSS
3 dpi
6 dpi
10 dpi

Supplement: Additional file 2: — Effect of iron concentration and fadD -loss-of function on the structure of S. meliloti GR4 biofilms. CLSM images showing the xy view of 3-, 6- and 10-day-old biofilms developed by GFP-labelled GR4 and fadD derivative mutant cells on chambered cover glass slides after growth in MM containing different iron concentrations. Both strains develop flat unstructured biofilms when they are grown in MM containing low iron concentration (22 μM FeCl3). In the presence of high iron conditions (220 μM FeCl3), the wild-type strain GR4 develops highly structured biofilms. The characteristic sponge-like structure of these biofilms is lost after 10 days, suggesting the initiation of biofilm dispersal. The fadD mutant (GR4FDCSS) is unable to develop structured biofilms even in the presence of iron-rich conditions. Bars, 15 μm. [file 12866_2015_390_MOESM2_ESM.pptx]
