# Supplementary material for: Biofilm formation assessment in Sinorhizobium meliloti reveals interlinked control with surface motility
Source: BMC Microbiol. 2015 Mar 3;15:58. doi: 10.1186/s12866-015-0390-z (PMC4381460; doi:10.1186/s12866-015-0390-z)
Supplement: Additional file 3: — Effect of iron concentration on fadD gene expression in S. meliloti GR4 and Rm1021. [file 12866_2015_390_MOESM3_ESM.docx]

Additional file 3 – Effect of iron concentration on *fadD* gene expression in *S. meliloti* GR4 and Rm1021

|  | β-Galactosidase activity (U)^a^ | | | |
| --- | --- | --- | --- | --- |
|  | *S. meliloti* GR4 | | *S. meliloti* Rm1021 | |
| Concentration of FeCl_3_ in MM | pGD499 (*npt::lacZ*) | pMPD4 (*fadD::lacZ*) | pGD499 (*npt::lacZ*) | pMPD4 (*fadD::lacZ*) |
| 2.2 µM | 314 ± 23 | 520 ± 43 * | 202 ± 15 | 331 ± 9 ** |
| 22 µM | 321 ± 48 | 419 ± 51 | 211 ± 4 | 220 ± 4 |
| 220 µM | 276 ± 23 | 310 ± 7 | 289 ± 40 | 227 ± 6 |

^a^Mean values and standard errors were calculated from two independent experiments. * indicates significant differences (P < 0.05) of *fadD* expression relative to 220 µM FeCl_3_ according to an ANOVA test. ** indicates significant differences (P < 0.01) of *fadD* expression relative to both 22 µM and 220 µM FeCl_3_ according to an ANOVA test.

Plasmid pGD499 [1] containing an *npt::lacZ* transcriptional fusion was used as control to test the expression of the constitutive kanamycin resistance gene. Plasmid pMPD4 is a pMP220 [2] derivative harboring a *fadD::lacZ* transcriptional fusion. To obtain pMPD4, a 0.6 kb *Eco*RI fragment containing the *fadD* promoter region, was isolated from cosmid pRmersf442 [3] and subcloned into pMP220. The correct orientation was checked with a *Kpn*I digestion. Plasmids pGD499 and pMPD4 were introduced into *S. meliloti* strains by a biparental mating using the *E. coli* mobilizing strain S17-1.

For measurement of β-galactosidase activity, *S. meliloti* cells containing *lacZ* fusions were grown in liquid MM containing different concentrations of FeCl_3_ (2.2, 22 or 220 µM). Samples of 100 µl of bacterial cultures were taken and assayed for β-galactosidase activity by the SDS-chloroform method described by Miller [4].

1. Ditta G, Schmidhauser T, Yakobson E, Lu P, Liang XW, Finlay DR, Guiney D, Helinski D: **Plasmids related to the broad host range vector, pRK290, useful for gene cloning and for monitoring gene expression.** *Plasmid* 1985, **13**:149-153

2. Spaink, HP, Okker RJH, Wijffelman CA, Pees E, Lugtenberg BJJ: **Promoters in the nodulation region of the *Rhizobium leguminosarum* Sym plasmid pRL1JI.** *Plant Mol Biol* 1987, **9**:27–39

3. Soto MJ, Fernández-Pascual M, Sanjuán J, Olivares J: **A *fadD* mutant of *Sinorhizobium meliloti* shows multicellular swarming migration and is impaired in nodulation efficiency on alfalfa roots**. *Mol Microbiol* 2002, **43**:371-382

4. Miller JH: *Experiments in Molecular Genetics.* Cold Spring Harbor Laboratory, Cold Spring Harbor, N.Y; 1972.
